# Synthesis, crystal structure and properties of *catena*-poly[[[bis­(3-methyl­pyridine-κ*N*)nickel(II)]-di-μ-1,3-thio­cyanato] aceto­nitrile monosolvate]

**DOI:** 10.1107/S2056989022009598

**Published:** 2022-10-06

**Authors:** Christian Näther, Inke Jess, Christoph Krebs

**Affiliations:** a Institut für Anorganische Chemie, Universität Kiel, Max-Eyth.-Str. 2, 24118 Kiel, Germany; Texas A & M University, USA

**Keywords:** crystal structure, solvate, nickel thio­cyanate, solvent removal

## Abstract

In the crystal structure of the title compound, {[Ni(NCS)_2_(C_6_H_7_N)_2_]·CH_3_CN}_*n*_, the Ni^II^ cation is octa­hedrally coordinated by two N-bonding and two S-bonding thio­cyanate anions, as well as two 3-methyl­pyridine coligands, with the thio­cyanate S atoms and the 3-methyl­pyridine N atoms in *cis*-positions. The metal cations are linked by pairs of thio­cyanate anions into chains that, because of the *cis*–*cis*–*trans* coordination, are corrugated. These chains are arranged in such a way that channels are formed in which disordered aceto­nitrile solvate mol­ecules are located. This overall structural motif is very similar to that observed in Ni(NCS)_2_[4-(boc-amino)­pyridine]_2_·CH_3_CN reported in the literature. At room temperature, the title compound loses its solvent mol­ecules within a few hours, leading to a crystalline phase that is structurally related to that of the pristine material. If the ansolvate is stored in an aceto­nitrile atmosphere, the solvate is formed again. Single-crystal X-ray analysis at room-temperature proves that the crystals decompose immediately, presumably because of the loss of solvent mol­ecules, and from the reciprocal space plots it is obvious that this reaction, in contrast to that in Ni(NCS)_2_[4-(boc-amino)­pyridine]_2_·CH_3_CN, does not proceed *via* a topotactic reaction.

## Abstract 2

In the crystal structure of the title compound, the Ni cations are octa­hedrally coordinated and linked into chains that are arranged in such a way that channels are formed in which aceto­nitrile solvate mol­ecules are embedded that can reversibly be removed.

## 1. Chemical context

Over the past several years, we and others have been inter­ested in the synthesis and crystal structures of coordination polymers based on transition-metal cations and thio­cyanate anions. For this anionic ligand, two major coordination modes are known, which include terminal coordination and the μ-1,3-bridging mode. The latter mode is of special inter­est if magnetic coordination polymers are to be prepared, because thio­cyanate anions can mediate reasonable magnetic exchange (Palion-Gazda *et al.*, 2015 ▸; Mekuimemba *et al.*, 2018 ▸; Böhme & Plass, 2019 ▸; Rams *et al.*, 2020 ▸). In the majority of such compounds, the metal cations are octa­hedrally coordin­ated by each of two *trans* thio­cyanate S and N atoms as well as two N atoms of neutral coligands that mostly consist of pyridine derivatives. The metal cations are linked by pairs of anionic ligands into chains that, because of the all-*trans* coordination, are linear (Shurdha *et al.*, 2013 ▸; Prananto *et al.*, 2017 ▸; Mautner, Traber *et al.*, 2018 ▸; Jochim *et al.*, 2020*a* ▸,*b* ▸).

For octa­hedrally coordinated metal cations, however, five different isomers exist, which include the all-*trans*, all-*cis* and three *cis*–*cis*–*trans* coordinations. For compounds based on thio­cyanate anions, the all-*trans* coordination is the most common, the all-*cis* coordination is unknown and the *cis*–*cis*–*trans*-coordination is very rare. It is noted that the latter coordination leads to the formation of linear chains if the coligands are in the *trans*-position (Werner *et al.*, 2014 ▸, 2015*a* ▸,*b* ▸), whereas corrugated chains are observed if they are in the *cis*-position (Böhme *et al.*, 2020 ▸; Suckert *et al.*, 2017 ▸).

**Figure 1:**
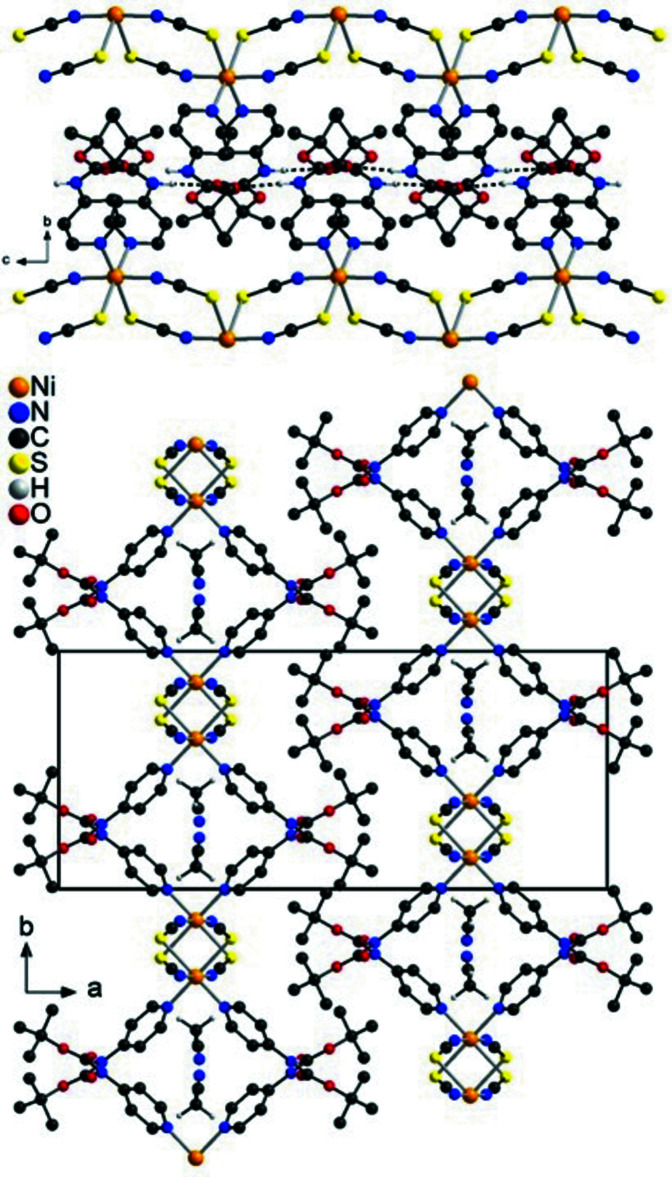
Crystal structure of di­thio­cyanato­bis­[4-(boc-amino)­pyridine]­nickel(II) aceto­nitrile solvate retrieved from the literature showing a view of the chains with inter­molecular N—H⋯O hydrogen bonding indicated by dashed lines (top) and with a view along the crystallographic *c*-axis (bottom).

In this context, we have reported on a compound with the composition Ni(NCS)_2_[4-(boc-amino)­pyridine]_2_·CH_3_CN in which the Ni^II^ cations are octa­hedrally coordinated by four μ-1,3-bridging thio­cyanate anions as well as two 4-(boc-amino)­pyridine ligands (Suckert *et al.*, 2017 ▸). The coligands and the S-bonding thio­cyanate anions are in *cis*-positions, whereas the two N-bonding anionic ligands are *trans*, leading to the formation of corrugated chains (Fig. 1 ▸: top). These chains are inter­connected by strong N—H⋯O hydrogen bonding into layers that are packed in such a way that channels are formed in which disordered aceto­nitrile solvate mol­ecules are located (Fig. 1 ▸: bottom). The aceto­nitrile mol­ecules can be removed under vacuum and reincorporated *via* the gas phase without any loss in crystallinity. More importantly, single-crystal structure analysis of one crystal showed that the solvent removal is accompanied by a change in symmetry from primitive to *C*-centered. If this crystal is stored in an aceto­nitrile atmosphere, the solvent is reincorporated and the reflections violating the *C*-centering are observed again. Images of reciprocal space at different aceto­nitrile contents look like that of a single crystal, but the mosaic spread increases during formation of the ansolvate and reformation of the solvate, which proves that these reactions proceed *via* a topotactic reaction (Suckert *et al.*, 2017 ▸).

In the course of our systematic work we became inter­ested in Ni(NCS)_2_ compounds based on 3-methyl­pyridine (3-picoline) as coligand. Many compounds have been reported with this ligand, but with nickel only discrete complexes with a terminal coordination are known and most of these compounds consist of solvates (see *Database survey*). An Ni(NCS)_2_ compound with 3-methyl­pyridine that shows a bridging coordination of the anionic ligands does not exist. 
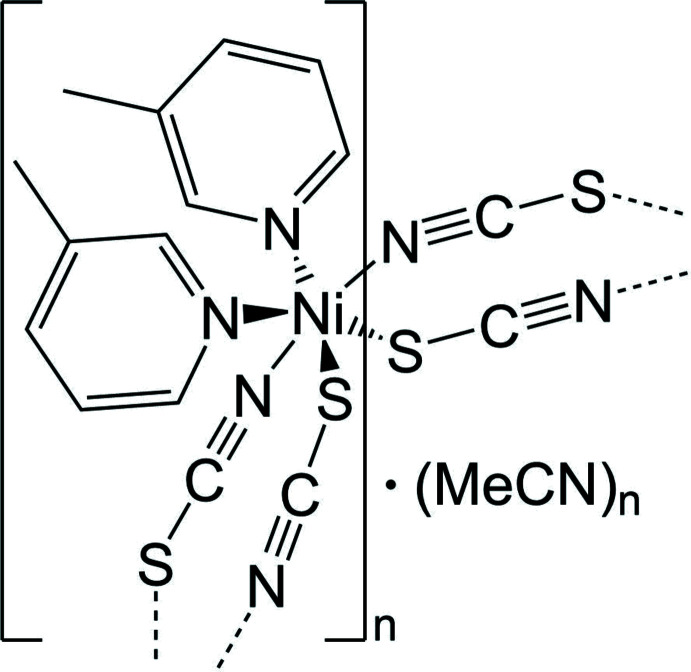


 However, in the course of our systematic investigations we accidentally obtained crystals of a further crystalline phase with the composition Ni(NCS)_2_(3-methyl­pyridine)_2_·aceto­nitrile. Single-crystal structure analysis shows that a network has formed, which is very similar to that observed in Ni(NCS)_2_[4-(boc-amino)­pyridine]_2_·aceto­nitrile mentioned above. That both compounds are structurally related is already obvious from their similar unit-cell parameters, but also from the crystal symmetry (see *Structural commentary*). X-ray powder diffraction proves the formation of a pure crystalline phase (Fig. S1 in the supporting information). In the IR spectrum, the CN-stretching vibration of the thio­cyanate anion is observed at 2109 cm^−1^, in agreement with the presence of μ-1,3-bridging thio­cyanate anions and that of the aceto­nitrile solvate mol­ecules at 2164 cm^−1^, proving the presence of aceto­nitrile (Fig. S2). In view of these results, we investigated whether the aceto­nitrile solvate mol­ecules can be removed from the title compound and if this proceeds *via* a topotactic reaction as observed in Ni(NCS)_2_[4-(boc-amino)­pyridine]_2_·CH_3_CN mentioned above (Suckert *et al.*, 2017 ▸). Experiments using X-ray powder diffraction show that the crystals have already decomposed at room temperature because of the loss of the solvate mol­ecules, leading to the formation of a crystalline phase. The IR spectrum is very similar to that of the pristine phase but the CN-stretching vibration of the aceto­nitrile ligands have disappeared, proving that the ansolvate has formed (Fig. S3). The X-ray powder pattern of the ansolvate obtained by storing the title compound for 24 h at room temperature is very similar to that of the pristine material, which indicates that both structures must be strongly related (Fig. S4). In particular, the first three intense reflections are shifted to higher Bragg angles, which is in agreement with a decrease of the unit-cell volume. If the ansolvate is stored for 3 d in a desiccator in an aceto­nitrile atmosphere, the powder pattern is identical to that calculated for the title compound, which proves that this process is reversible. We also tried to determine the crystal structure of the title compound at room temperature, but during the measurement the crystal started to decompose and no reasonable data were obtained. However, the lattice parameters were determined from indexing the reflections and used for the calculation of the powder patterns. Moreover, from the reciprocal space plots of this data set, it is obvious that the mosaic spread strongly increases, which would be in agreement with a topotactic reaction, but the diffraction pattern does not look like that of a single crystal, as was the case for Ni(NCS)_2_[4-(boc-amino)­pyridine]_2_·CH_3_CN mentioned above (Suckert *et al.*, 2017 ▸).

## 2. Structural commentary

**Figure 2:**
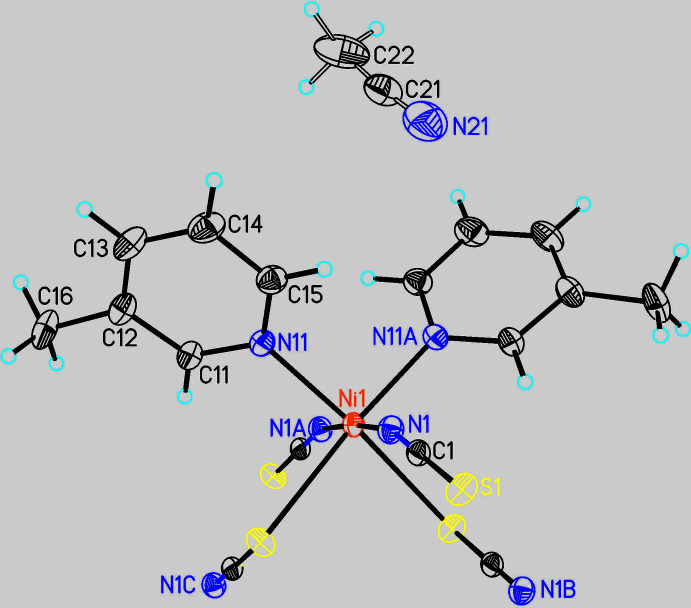
Crystal structure of the title compound with labeling and displacement parameters drawn at the 50% probability level. Symmetry codes: (A) −*x* + 1, *y*, −*z* + 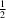 ; (B) −*x* + 1, −*y*, −*z*; (C) *x*, −*y*, *z* + 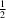 .

The asymmetric unit of the title compound consists of one Ni^II^ cation, two thio­cyanate anions, two 3-methyl­pyridine ligands and one aceto­nitrile mol­ecule, all of them located in general positions (Fig. 2 ▸). The Ni cations are octa­hedrally coordinated by two 3-methyl­pyridine coligands and two N- as well two S-bonding thio­cyanate anions in a *cis*–*cis*–*trans* coordination with the thio­cyanate S atoms and the 3-methyl­pyridine N atoms in *cis*-positions. The Ni—N and Ni—S bond lengths correspond to those in similar compounds (Table 1 ▸). From the bonding angles, it is obvious that the octa­hedra are slightly distorted (Table 1 ▸). This is also obvious from the values of the octa­hedral angle variance and the mean octa­hedral quadratic elongation calculated by the method of Robinson *et al.* (1971 ▸), which amount to 12.7996 and 1.0190.

**Figure 3:**
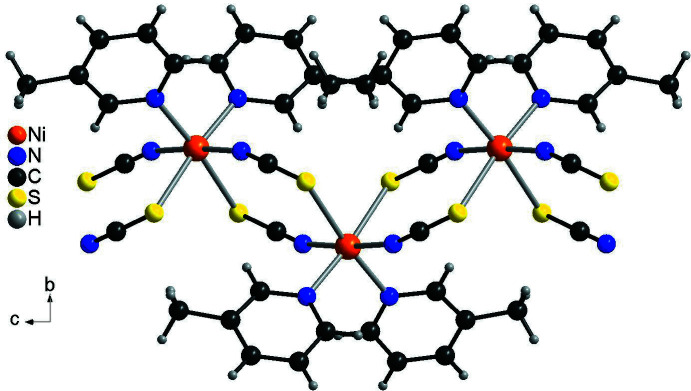
Crystal structure of the title compound with view of part of a chain showing the Ni coordination along the crystallographic *a*-axis.

The metal cations are linked by pairs of anionic ligands into chains that are corrugated because of the *cis*-coordination of the 3-methyl­pyridine ligands (Fig. 3 ▸).

## 3. Supra­molecular features

**Figure 4:**
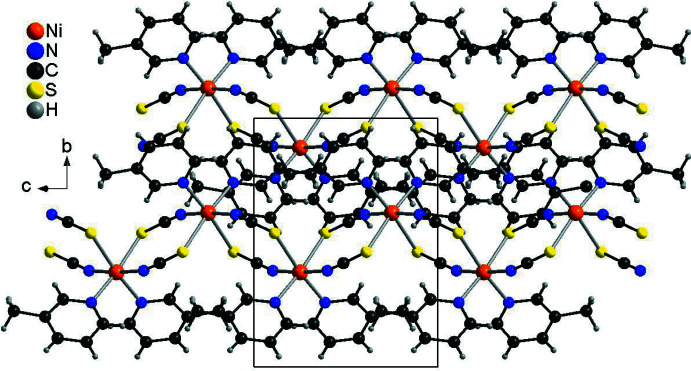
Crystal structure of the title compound with view along the crystallographic *a*-axis.

In the crystal structure of the title compound, the chains proceed in the direction of the crystallographic *c*-axis and are arranged in such a way that cavities are formed, in which disordered aceto­nitrile mol­ecules are embedded (Figs. 4 ▸ and 5 ▸). This arrangement is very similar to that observed in Ni(NCS)_2_[4-(boc-amino)­pyridine]_2_·CH_3_CN already reported in the literature (please compare Fig. 1 ▸ with Figs. 4 ▸ and 5 ▸, Suckert *et al.*, 2017 ▸). That this structure is structurally related to that of the title compound is also indicated by comparing their unit-cell parameters and their space groups. Ni(NCS)_2_[4-(boc-amino)­pyridine]_2_·CH_3_CN crystallizes in space group *P*2_1_/*n* with *a* = 26.5715 (7) Å, *b* = 11.4534 (4) Å, *c* = 9.8286 (2) Å and β = 94.982 (2)°, whereas the corresponding ansolvate crystallizes in space group *C*2/*c* with *a* = 26.7251 (8) Å, *b* = 11.3245 (5) Å, *c* = 9.8036 (3) Å and β = 94.922 (2)°. For a better comparison of the crystal structure of the title compound with that of Ni(NCS)_2_[4-(boc-amino)­pyridine]_2_·CH_3_CN already reported in the literature, the unit-cell parameters of the title compound must be given for the unconventional setting *I*2/*c*, leading to values of *a* = 16.3513 (1) Å, *b* = 11.7493 (1) Å, *c* = 9.7383 (1) Å and β = 94.9271 (1)°. The much larger value of the *a*-axis in the 4-(boc-amino)­pyridine compound originates from the much larger size of this neutral coligand, separating the Ni(NCS)_2_ chains more effectively.

Finally, it is noted that there are no pronounced inter­molecular hydrogen bonds in the title compound, except for one C—H⋯N contact that is much too long for any significant inter­action [C22—H22*A*⋯N21(1 − *x*, *y*, 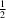 − *z*), H⋯N = 1.61 Å, C⋯N = 2.57 (2) Å, C—H⋯N = 162°]. This is in contrast to Ni(NCS)_2_[4-(boc-amino)­pyridine]_2_·CH_3_CN where the chains are linked by strong N—H⋯O hydrogen bonding, which might be the reason why this compound is much more stable than the title compound.

## 4. Database survey

A search in the Cambridge Structure Database (CSD, version 5.43, last update November 2021; Groom *et al.*, 2016 ▸) for transition-metal thio­cyanate compounds with 3-methyl­pyridine as coligand leads to several hits. There are a couple of known compounds containing nickel, all of which are discrete complexes of the composition Ni(NCS)_2_(3-methyl­pyridine)_4_ that contain additional solvate mol­ecules such as one mol­ecule per complex of a mixture of di­bromo and di­chloro­methane, of 2,2-di­chloro­propane and of di­chloro­methane, as well as two mol­ecules of di­chloro­methane and tri­chloro­methane (LAYLAY, LAYLEC, LAYLUS, LAYLIG and LAYLOM; Pang *et al.*, 1992 ▸). Moreover, crystal structures of the mono-tri­chloro­methane (CIVJEW and CIFJEW01; Nassimbeni *et al.*, 1984 ▸, 1986 ▸) and mono­tetra­chloro­methane solvate (JICMIR; Pang *et al.*, 1990 ▸) have also been reported. In Ni(NCS)_2_(3-methyl­pyridine)_2_(H_2_O)_2_, two of the coligands are substituted by aqua ligands and no solvate mol­ecules are present (MEGCEH; Tan *et al.*, 2006 ▸).

In the discrete copper complex Cu(NCS)_2_(3-methyl­pyridine)_2_ (ABOTET; Handy *et al.*, 2017 ▸) the metal center is fourfold and in Cu(NCS)_2_(3-methyl­pyridine)_3_ (VEPBAT; Kabešová & Kožíšková, 1989 ▸) fivefold coordinated. Also one more copper compound with the composition Cu(NCS)(3-methyl­pyridine)_2_ (CUHBEM; Healy *et al.*, 1984 ▸) has been reported in which the cations are tetra­hedrally coordinated by two coligands and also two thio­cyanate anions, linking them into chains. Some compounds with Co(NCS)_2_ and 3-methyl­pyridine can also be found in the CSD. All are discrete complexes, but Co(NCS)_2_(3-methyl­pyridine)_2_ (EYARIG; Boeckmann *et al.*, 2011 ▸) has a tetra­hedral coordination around the metal center compared to the octa­hedral complexes Co(NCS)_2_(3-methyl­pyridine)_4_ (EYAROM and EYAROM01; Boeckmann *et al.*, 2011 ▸ and Małecki *et al.*, 2012 ▸) and Co(NCS)_2_(3-methyl­pyridine)_2_(H_2_O)_2_ (EYAREC; Boeckmann *et al.*, 2011 ▸).

With zinc and cadmium, just one compound could be found each, *viz*. the discrete tedrahedral complex Zn(NCS)_2_(3-methyl­pyridine)_2_ (ETUSAO; Boeckmann & Näther, 2011 ▸) and Cd(NCS)_2_(3-methyl­pyridine)_2_ (FIYGUP; Taniguchi *et al.*, 1987 ▸) with octa­hedrally coordinated cations that are linked into chains by the thio­cyanate anions. Although not yet included in this CSD version, an octa­hedral iron complex is known, with the cations coordinated by two thio­cyanate anions and four 3-methyl­pyridine ligands (Ceglarska *et al.*, 2022 ▸), which was reported analogously also as an isotypic complex with manganese in the same publication. Otherwise, only one more manganese compound is reported, which however contains 3-methyl­pyridine-*N*-oxide coligands and consists of a chain structure (KESSAF; Mautner, Berger *et al.*, 2018 ▸). There are also two compounds with a mixed-metal composition, on the one hand with *catena*-[tetra­kis(thio­cyan­ato)­bis­(3-methyl­pyridine)­manganesemercury] (NAQYOW; Małecki, 2017*a* ▸) and on the other hand with *catena*-[tetra­kis(μ-thio­cyanato)­bis­(3-methyl­pyridine)­mercuryzinc] (QAM­SIJ; Mał­ecki, 2017*b* ▸).

## 5. Synthesis and crystallization

**Synthesis**

Ni(NCS)_2_ was purchased from Santa Cruz Biotechnology and 3-methyl­pyridine was purchased from Alfa Aesar. Aceto­nitrile, which was used as the solvent, was dried over CaH_2_ before use.

Ni(NCS)_2_(3-methyl­pyridine)_2_·aceto­nitrile: The reaction mixture containing 0.25 mmol of Ni(NCS)_2_ (43.7 mg) and 0.25 mmol of 3-methyl­pyridine (24.3 µl) in 1.5 mL of aceto­nitrile was stored for 2 days at room temperature, resulting in light-green crystals suitable for single-crystal X-ray diffraction measurements.

**Experimental details**

The data collection for single-crystal structure analysis was performed using an XtaLAB Synergy, Dualflex, HyPix diffractometer from Rigaku with Cu *K*α radiation. The PXRD measurements were performed with a Stoe Transmission Powder Diffraction System (STADI P) that is equipped with a MYTHEN 1K detector and a Johansson-type Ge(111) monochromator using Cu *K*α_1_ radiation (λ = 1.540598 Å). The IR spectra were measured using an ATI Mattson Genesis Series FTIR Spectrometer, control software: *WINFIRST*, from ATI Mattson. The instruments were calibrated using standard reference materials.

## 6. Refinement

Crystal data, data collection and structure refinement details are summarized in Table 2 ▸. All non-hydrogen atoms were refined anisotropically. The C-bound H atoms were positioned with idealized geometry (methyl H atoms allowed to rotate but not to tip) and were refined isotropically with *U*_iso_(H) = 1.2*U*_eq_(C) (1.5 for methyl H atoms) using a riding model. The aceto­nitrile solvate mol­ecules are disordered within the channels around a center of inversion, which is located in the middle of two aceto­nitrile N atoms that show an N—N distance of 1.151 Å. Therefore, they were refined with an sof of 0.5, leading to reasonable anisotropic displacement parameters. The situation is similar to that in Ni(NCS)_2_[4-(boc-amino)­pyridine]_2_·aceto­nitrile mentioned above.

It is noted that some additional reflections are observed, leading to a doubling of the unit cell and change from *C*-centered to primitive. The relation between the sub-cell and the super cell is obvious if the super cell [*a* = 16.3542 (5) Å, *b* = 23.4916 (8) Å, *c* = 9.7358 (3) Å and β = 94.977 (3)°, space group *P*2_1_/*c*] is compared with the sub-cell in space group *I*2/*a* instead of *C*2/*c* [*a* = 9.7383 (1) Å, *b* = 11.7493 (1) Å, *c* = 16.3513 (1) Å and β = 94.927 (1)°]. However, only very few reflections were observed and their intensity is close to zero (Fig. S5). Nevertheless, the structure can easily be refined in space group *P*2_1_/*c*, leading to two crystallographically independent Ni^II^ cations and two unique aceto­nitrile ligands, but a closer look reveals that even in the super cell the solvate mol­ecules are disordered. Therefore, the very few and weak additional reflections were neglected.

## Supplementary Material

Crystal structure: contains datablock(s) I. DOI: 10.1107/S2056989022009598/jy2022sup1.cif

Structure factors: contains datablock(s) I. DOI: 10.1107/S2056989022009598/jy2022Isup2.hkl

Experimental (top) and calculated (bottom) X-ray powder pattern of the title compound. DOI: 10.1107/S2056989022009598/jy2022sup3.png

IR spectra of the title compound. The values of the CN stretching vibration of the thiocyanate anions and of the acetonitrile molecules are given. DOI: 10.1107/S2056989022009598/jy2022sup4.png

IR spectra of the title compound after 2h in air atmosphere. The value of the CN stretching vibration is given. DOI: 10.1107/S2056989022009598/jy2022sup5.png

Experimental X-ray powder pattern of the title compound stored for 24h in air atmosphere (top), of the ansolvate stored for 5d in an acetonitrile atmosphere (mid) and the calculated powder pattern (bottom). DOI: 10.1107/S2056989022009598/jy2022sup6.png

View of the reciprocal space in c* direction of a crystal of the title compound measured at room-temperature. DOI: 10.1107/S2056989022009598/jy2022sup7.png

CCDC reference: 2210399

Additional supporting information:  crystallographic information; 3D view; checkCIF report

## Figures and Tables

**Figure 5:**
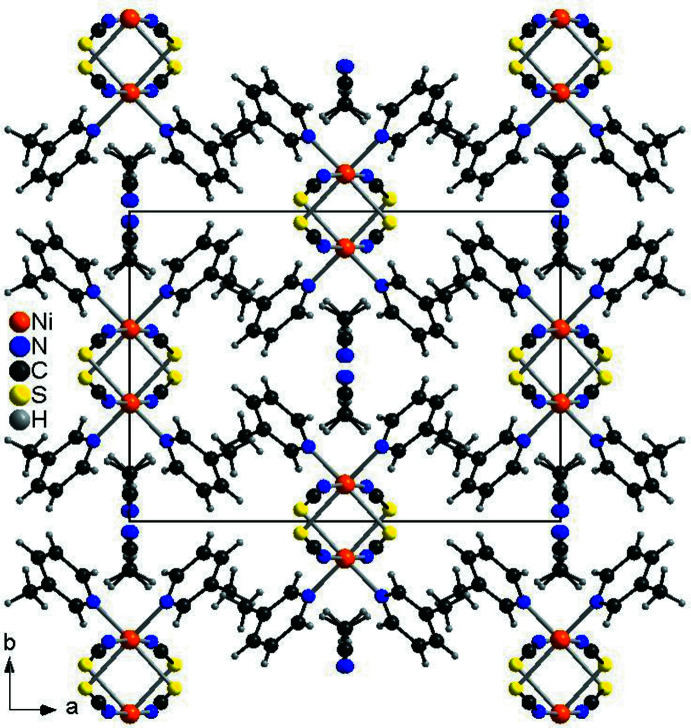
Crystal structure of the title compound with view along the crystallographic *c*-axis.

**Table 1:** Selected geometric parameters (Å, °)

Ni1—N1	2.0285 (11)	Ni1—S1^ii^	2.5508 (4)
Ni1—N1^i^	2.0286 (11)	Ni1—N11	2.0836 (11)
			
N1—Ni1—N1^i^	175.54 (6)	S1^iii^—Ni1—S1^ii^	87.022 (18)
N1—Ni1—S1^ii^	83.43 (3)	N11—Ni1—S1^iii^	173.74 (3)
N1—Ni1—S1^iii^	93.32 (3)	N11—Ni1—S1^ii^	89.87 (3)
N1—Ni1—N11	91.71 (4)	N11—Ni1—N11^i^	93.73 (6)
N1—Ni1—N11^i^	91.34 (4)		

Symmetry codes: (i) 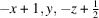 ; (ii) 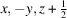 ; (iii) 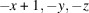 .

**Table 2:** Experimental details

Crystal data	
Chemical formula	[Ni(NCS)_2_(C_6_H_7_N)_2_]·C_2_H_3_N
*M*_r_	402.17
Crystal system, space group	Monoclinic, *C*2/*c*
Temperature (K)	100
*a*, *b*, *c* (Å)	18.2934 (9), 11.7472 (4), 9.7341 (5)
β (°)	117.043 (6)
*V* (Å^3^)	1863.11 (17)
*Z*	4
Radiation type	Cu *K*α
μ (mm^−1^)	3.65
Crystal size (mm)	0.15 × 0.15 × 0.03

Data collection	
Diffractometer	XtaLAB Synergy, Dualflex, HyPix
Absorption correction	Multi-scan (*CrysAlis PRO*; Rigaku OD, 2021 ▸)
*T*_min_, *T*_max_	0.705, 1.000
No. of measured, independent and observed [*I* > 2σ(*I*)] reflections	11840, 1966, 1955
*R*_int_	0.015

Refinement	
*R*[*F* ^2^ > 2σ(*F* ^2^)], *wR*(*F* ^2^), *S*	0.024, 0.066, 1.07
No. of reflections	1966
No. of parameters	125
H-atom treatment	H-atom parameters constrained
Δρ_max_, Δρ_min_ (e Å^−3^)	0.27, −0.37

Computer programs: *CrysAlis PRO* (Rigaku OD, 2021 ▸), *SHELXT2014/5* (Sheldrick, 2015*a* ▸), *SHELXL2016/6* (Sheldrick, 2015*b* ▸), *DIAMOND* (Brandenburg & Putz, 1999 ▸) and *publCIF* (Westrip, 2010 ▸).

## supplementary crystallographic information

### Crystal data

**Table:**

[Ni(NCS)_2_(C_6_H_7_N)_2_]·C_2_H_3_N	*F*(000) = 832
*M**_r_* = 402.17	*D*_x_ = 1.434 Mg m^−^^3^
Monoclinic, *C*2/*c*	Cu *K*α radiation, λ = 1.54178 Å
*a* = 18.2934 (9) Å	Cell parameters from 9752 reflections
*b* = 11.7472 (4) Å	θ = 4.6–77.2°
*c* = 9.7341 (5) Å	µ = 3.65 mm^−^^1^
β = 117.043 (6)°	*T* = 100 K
*V* = 1863.11 (17) Å^3^	Plate, light green
*Z* = 4	0.15 × 0.15 × 0.03 mm

### Data collection

**Table:**

XtaLAB Synergy, Dualflex, HyPix diffractometer	1966 independent reflections
Radiation source: micro-focus sealed X-ray tube, PhotonJet (Cu) X-ray Source	1955 reflections with *I* > 2σ(*I*)
Mirror monochromator	*R*_int_ = 0.015
Detector resolution: 10.0000 pixels mm^-1^	θ_max_ = 77.7°, θ_min_ = 4.6°
ω scans	*h* = −23→21
Absorption correction: multi-scan (CrysAlisPro; Rigaku OD, 2021)	*k* = −13→14
*T*_min_ = 0.705, *T*_max_ = 1.000	*l* = −10→12
11840 measured reflections	

### Refinement

**Table:**

Refinement on *F*^2^	Primary atom site location: dual
Least-squares matrix: full	Hydrogen site location: inferred from neighbouring sites
*R*[*F*^2^ > 2σ(*F*^2^)] = 0.024	H-atom parameters constrained
*wR*(*F*^2^) = 0.066	*w* = 1/[σ^2^(*F*_o_^2^) + (0.0356*P*)^2^ + 1.9913*P*] where *P* = (*F*_o_^2^ + 2*F*_c_^2^)/3
*S* = 1.07	(Δ/σ)_max_ = 0.001
1966 reflections	Δρ_max_ = 0.27 e Å^−^^3^
125 parameters	Δρ_min_ = −0.37 e Å^−^^3^
0 restraints	

### Special details

**Table:**

Geometry. All esds (except the esd in the dihedral angle between two l.s. planes) are estimated using the full covariance matrix. The cell esds are taken into account individually in the estimation of esds in distances, angles and torsion angles; correlations between esds in cell parameters are only used when they are defined by crystal symmetry. An approximate (isotropic) treatment of cell esds is used for estimating esds involving l.s. planes.

### Fractional atomic coordinates and isotropic or equivalent isotropic displacement parameters (Å^2^)

**Table:**

	*x*	*y*	*z*	*U*_iso_*/*U*_eq_	Occ. (<1)
Ni1	0.500000	0.11950 (2)	0.250000	0.01509 (10)	
N1	0.54965 (7)	0.11278 (9)	0.10148 (12)	0.0185 (2)	
C1	0.57231 (7)	0.08251 (10)	0.01406 (14)	0.0165 (2)	
S1	0.60344 (2)	0.03798 (3)	−0.11091 (4)	0.01977 (10)	
N11	0.58540 (6)	0.24076 (9)	0.38595 (12)	0.0181 (2)	
C11	0.62454 (8)	0.22828 (11)	0.53994 (15)	0.0204 (3)	
H11	0.607248	0.168907	0.584775	0.025*	
C12	0.68901 (8)	0.29758 (12)	0.63729 (16)	0.0244 (3)	
C13	0.71270 (9)	0.38429 (13)	0.56974 (18)	0.0297 (3)	
H13	0.756660	0.433386	0.631780	0.036*	
C14	0.67207 (10)	0.39913 (13)	0.41148 (19)	0.0309 (3)	
H14	0.687354	0.458893	0.363972	0.037*	
C15	0.60876 (9)	0.32557 (12)	0.32326 (16)	0.0240 (3)	
H15	0.581066	0.335802	0.214608	0.029*	
C16	0.73170 (9)	0.27472 (15)	0.80794 (17)	0.0336 (3)	
H16A	0.696992	0.226009	0.835783	0.050*	
H16B	0.741856	0.346916	0.864123	0.050*	
H16C	0.784063	0.236346	0.835127	0.050*	
N21	0.4958 (3)	0.5343 (3)	0.0380 (4)	0.0525 (8)	0.5
C21	0.4990 (2)	0.5882 (3)	0.1369 (4)	0.0366 (7)	0.5
C22	0.4981 (19)	0.6557 (4)	0.238 (3)	0.059 (3)	0.5
H22A	0.511446	0.613590	0.333381	0.089*	0.5
H22B	0.443345	0.689575	0.200183	0.089*	0.5
H22C	0.538683	0.716083	0.258413	0.089*	0.5

### Atomic displacement parameters (Å^2^)

**Table:**

	*U*^11^	*U*^22^	*U*^33^	*U*^12^	*U*^13^	*U*^23^
Ni1	0.01446 (16)	0.01706 (17)	0.01370 (16)	0.000	0.00635 (12)	0.000
N1	0.0176 (5)	0.0201 (5)	0.0177 (5)	−0.0016 (4)	0.0080 (4)	−0.0005 (4)
C1	0.0134 (5)	0.0164 (6)	0.0163 (6)	−0.0017 (4)	0.0039 (4)	0.0010 (4)
S1	0.01886 (16)	0.02257 (18)	0.02138 (17)	−0.00378 (11)	0.01220 (13)	−0.00468 (11)
N11	0.0172 (5)	0.0192 (5)	0.0184 (5)	−0.0010 (4)	0.0084 (4)	−0.0021 (4)
C11	0.0190 (6)	0.0237 (6)	0.0189 (6)	−0.0030 (5)	0.0087 (5)	−0.0027 (5)
C12	0.0199 (6)	0.0288 (7)	0.0245 (6)	−0.0049 (5)	0.0101 (5)	−0.0068 (5)
C13	0.0275 (7)	0.0305 (8)	0.0324 (8)	−0.0124 (6)	0.0148 (6)	−0.0105 (6)
C14	0.0358 (8)	0.0268 (7)	0.0348 (8)	−0.0101 (6)	0.0203 (7)	−0.0018 (6)
C15	0.0271 (6)	0.0223 (6)	0.0245 (6)	−0.0022 (5)	0.0135 (5)	−0.0006 (5)
C16	0.0284 (7)	0.0454 (9)	0.0219 (7)	−0.0141 (7)	0.0070 (6)	−0.0098 (6)
N21	0.071 (2)	0.0457 (19)	0.0431 (19)	−0.0022 (17)	0.0280 (17)	0.0091 (14)
C21	0.0440 (19)	0.0299 (16)	0.0381 (18)	0.0031 (14)	0.0206 (15)	0.0091 (15)
C22	0.080 (3)	0.0398 (18)	0.075 (7)	−0.004 (6)	0.050 (4)	0.017 (6)

### Geometric parameters (Å, º)

**Table:**

Ni1—N1	2.0285 (11)	C13—H13	0.9500
Ni1—N1^i^	2.0286 (11)	C13—C14	1.384 (2)
Ni1—S1^ii^	2.5508 (4)	C14—H14	0.9500
Ni1—S1^iii^	2.5508 (4)	C14—C15	1.386 (2)
Ni1—N11^i^	2.0836 (11)	C15—H15	0.9500
Ni1—N11	2.0836 (11)	C16—H16A	0.9800
N1—C1	1.1590 (17)	C16—H16B	0.9800
C1—S1	1.6456 (13)	C16—H16C	0.9800
N11—C11	1.3435 (16)	N21—C21	1.132 (5)
N11—C15	1.3360 (17)	C21—C22	1.270 (19)
C11—H11	0.9500	C22—H22A	0.9800
C11—C12	1.3912 (18)	C22—H22B	0.9800
C12—C13	1.384 (2)	C22—H22C	0.9800
C12—C16	1.504 (2)		
			
N1—Ni1—N1^i^	175.54 (6)	C13—C12—C11	117.29 (13)
N1—Ni1—S1^iii^	83.43 (3)	C13—C12—C16	122.58 (13)
N1^i^—Ni1—S1^iii^	93.32 (3)	C12—C13—H13	120.1
N1—Ni1—S1^ii^	93.32 (3)	C14—C13—C12	119.72 (13)
N1^i^—Ni1—S1^ii^	83.43 (3)	C14—C13—H13	120.1
N1—Ni1—N11	91.71 (4)	C13—C14—H14	120.5
N1—Ni1—N11^i^	91.34 (4)	C13—C14—C15	119.06 (13)
N1^i^—Ni1—N11	91.34 (4)	C15—C14—H14	120.5
N1^i^—Ni1—N11^i^	91.71 (4)	N11—C15—C14	122.18 (13)
S1^ii^—Ni1—S1^iii^	87.022 (18)	N11—C15—H15	118.9
N11^i^—Ni1—S1^iii^	173.74 (3)	C14—C15—H15	118.9
N11—Ni1—S1^ii^	173.74 (3)	C12—C16—H16A	109.5
N11^i^—Ni1—S1^ii^	89.87 (3)	C12—C16—H16B	109.5
N11—Ni1—S1^iii^	89.87 (3)	C12—C16—H16C	109.5
N11—Ni1—N11^i^	93.73 (6)	H16A—C16—H16B	109.5
C1—N1—Ni1	163.89 (10)	H16A—C16—H16C	109.5
N1—C1—S1	179.13 (12)	H16B—C16—H16C	109.5
C1—S1—Ni1^ii^	101.56 (4)	N21—C21—C22	174.4 (12)
C11—N11—Ni1	119.96 (9)	C21—C22—H22A	109.5
C15—N11—Ni1	121.53 (9)	C21—C22—H22B	109.5
C15—N11—C11	118.17 (11)	C21—C22—H22C	109.5
N11—C11—H11	118.2	H22A—C22—H22B	109.5
N11—C11—C12	123.56 (12)	H22A—C22—H22C	109.5
C12—C11—H11	118.2	H22B—C22—H22C	109.5
C11—C12—C16	120.10 (13)		
			
Ni1—N11—C11—C12	172.20 (10)	C11—C12—C13—C14	0.4 (2)
Ni1—N11—C15—C14	−172.51 (11)	C12—C13—C14—C15	−0.8 (2)
N11—C11—C12—C13	0.6 (2)	C13—C14—C15—N11	0.2 (2)
N11—C11—C12—C16	−177.52 (13)	C15—N11—C11—C12	−1.20 (19)
C11—N11—C15—C14	0.8 (2)	C16—C12—C13—C14	178.47 (15)

Symmetry codes: (i) −*x*+1, *y*, −*z*+1/2; (ii) −*x*+1, −*y*, −*z*; (iii) *x*, −*y*, *z*+1/2.
