# Supplementary figures and images for: Synthesis, crystal structure and properties of catena-poly[[[bis­(3-methyl­pyridine-κN)nickel(II)]-di-μ-1,3-thio­cyanato] aceto­nitrile monosolvate]
Source: Acta Crystallogr E Crystallogr Commun. 2022 Oct 6;78(Pt 11):1097–102. doi: 10.1107/S2056989022009598 (PMC9638983; doi:10.1107/S2056989022009598)

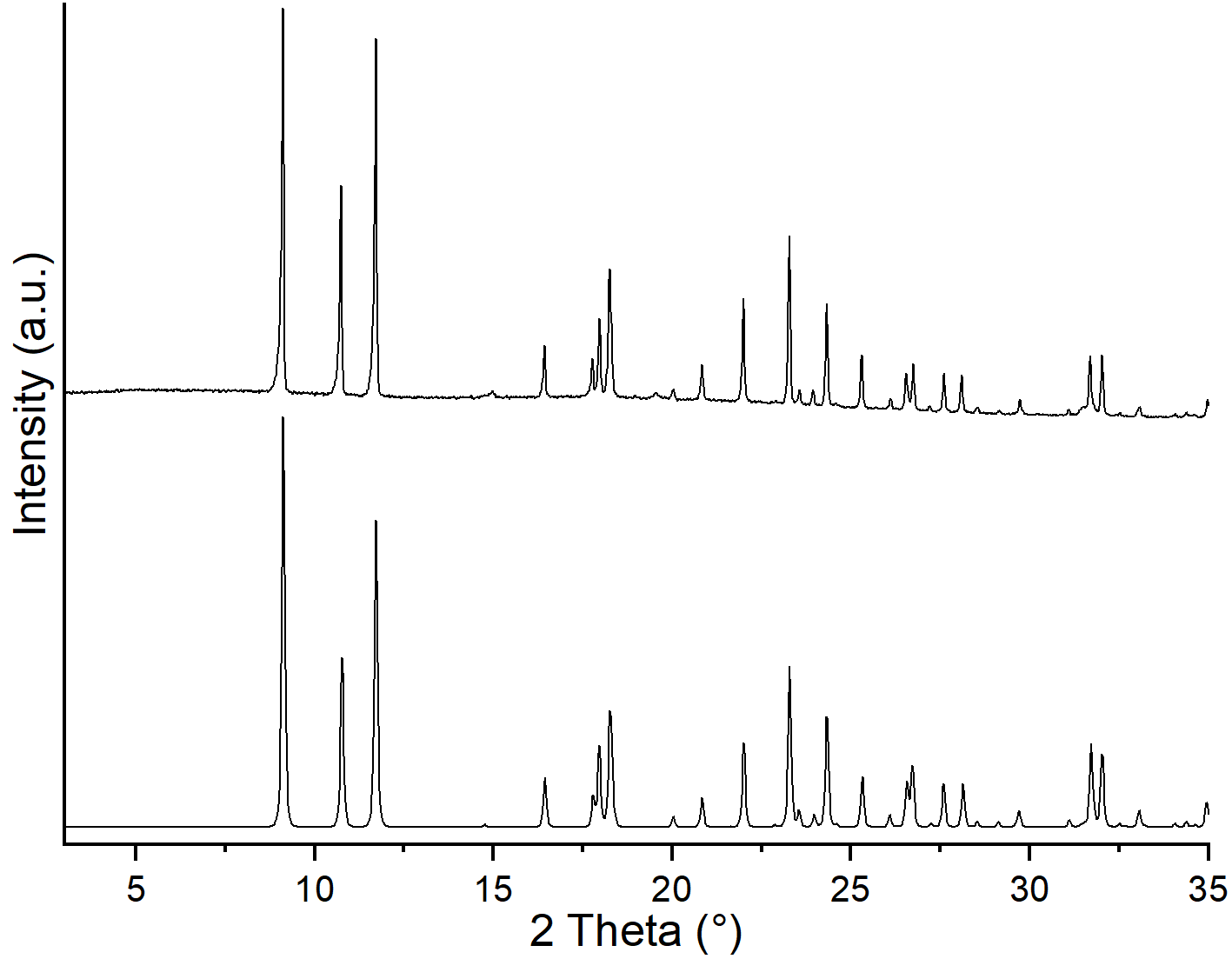

Supplement: Supplementary file 3 [file e-78-01097-sup3.png]

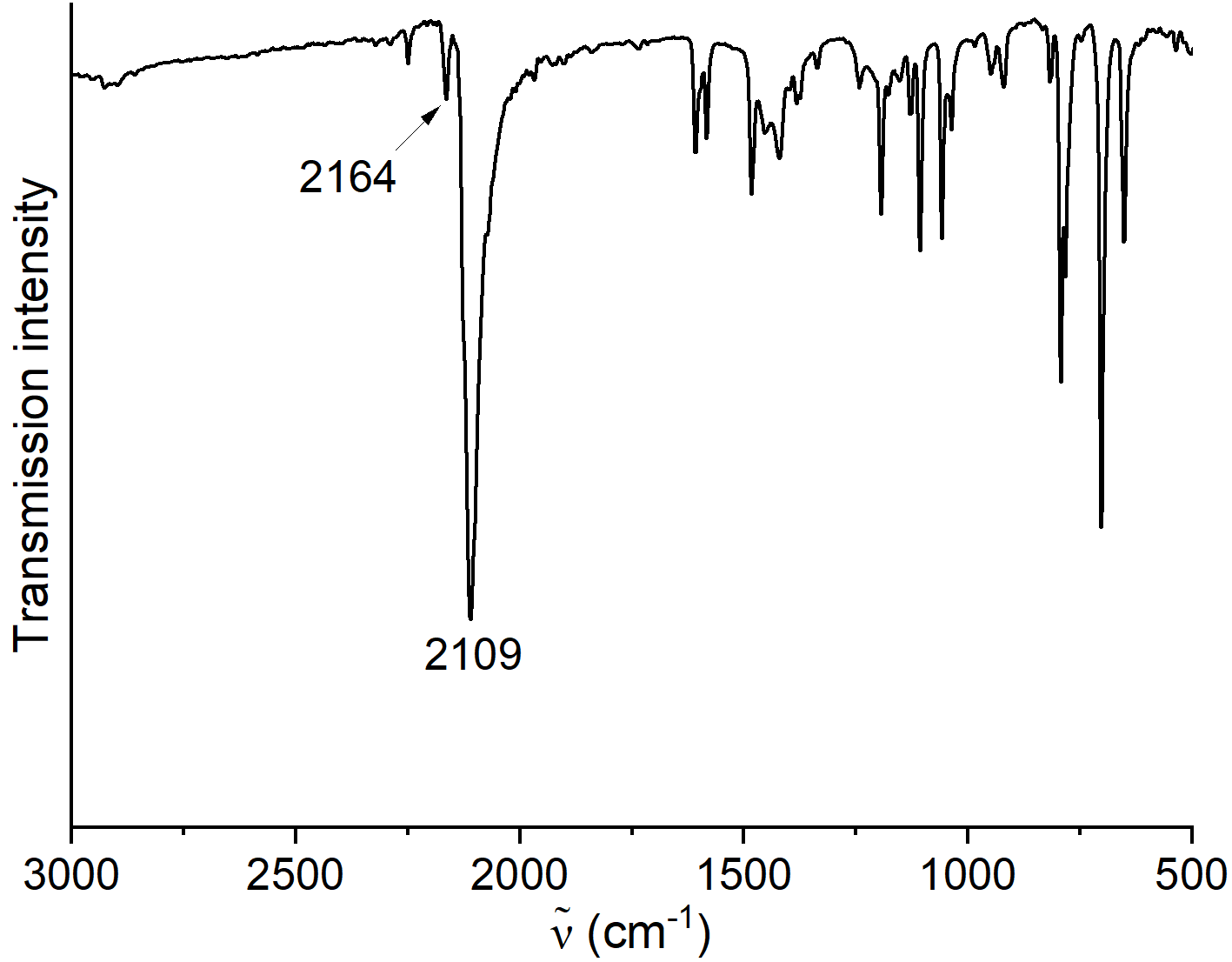

Supplement: Supplementary file 4 [file e-78-01097-sup4.png]

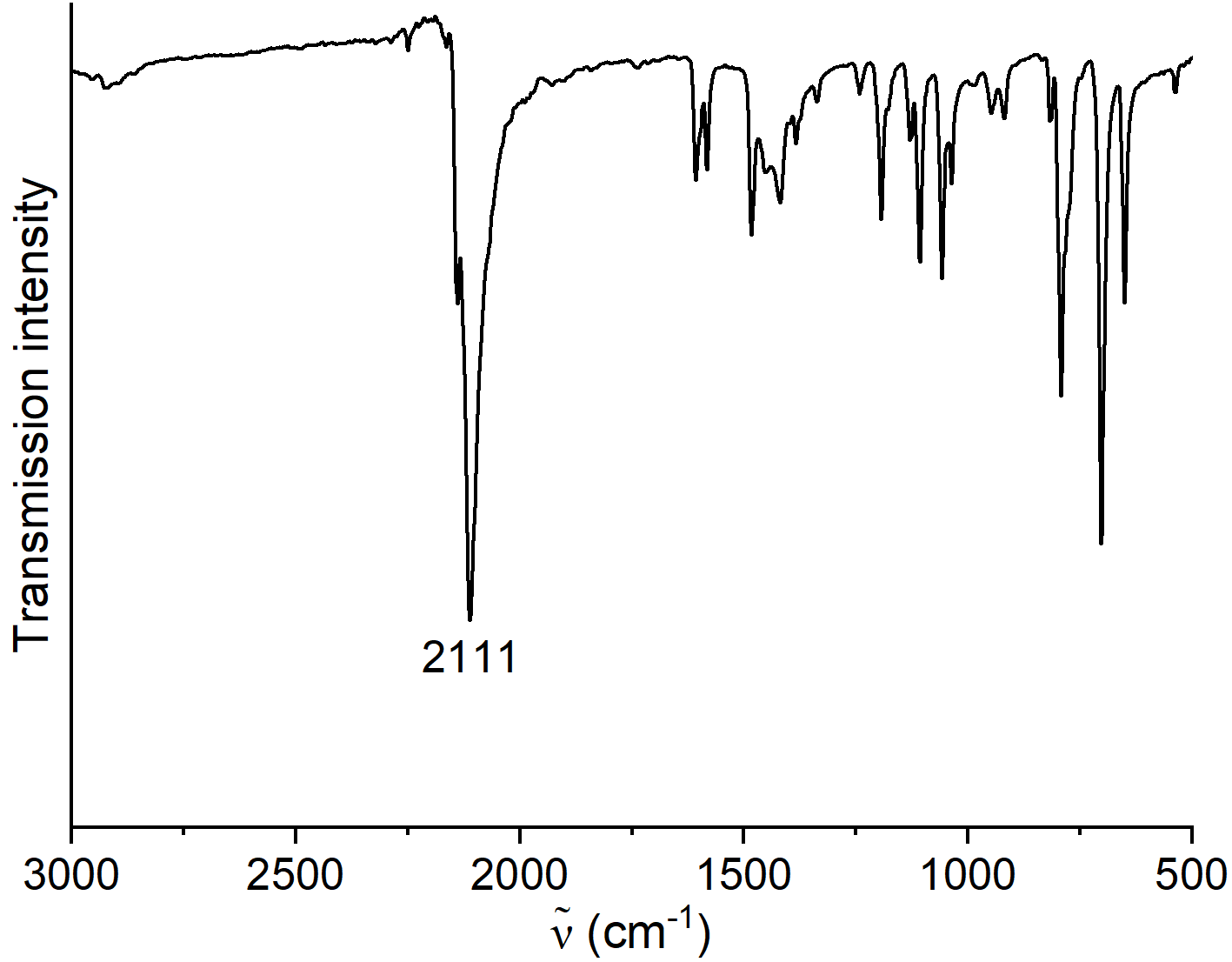

Supplement: Supplementary file 5 [file e-78-01097-sup5.png]

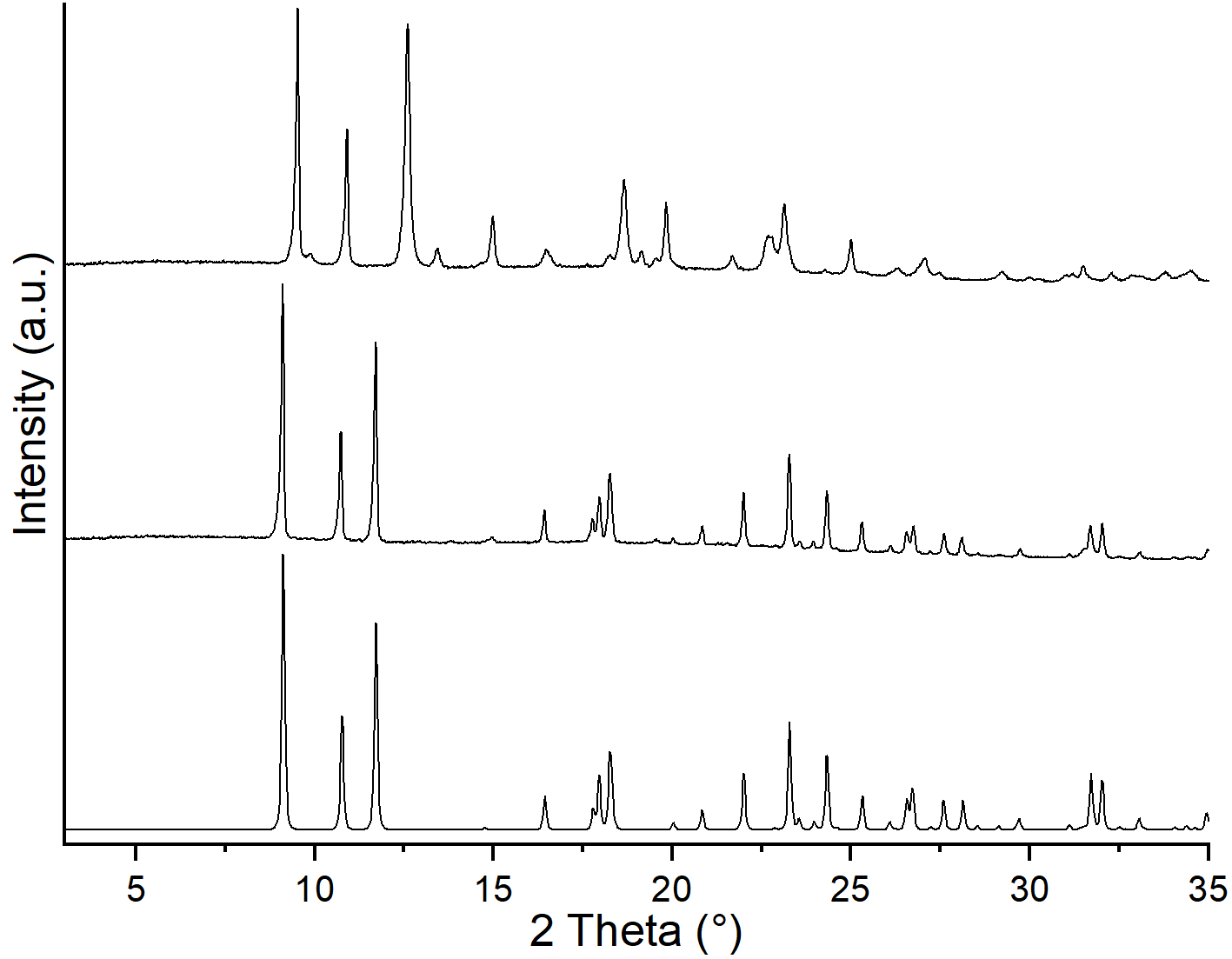

Supplement: Supplementary file 6 [file e-78-01097-sup6.png]

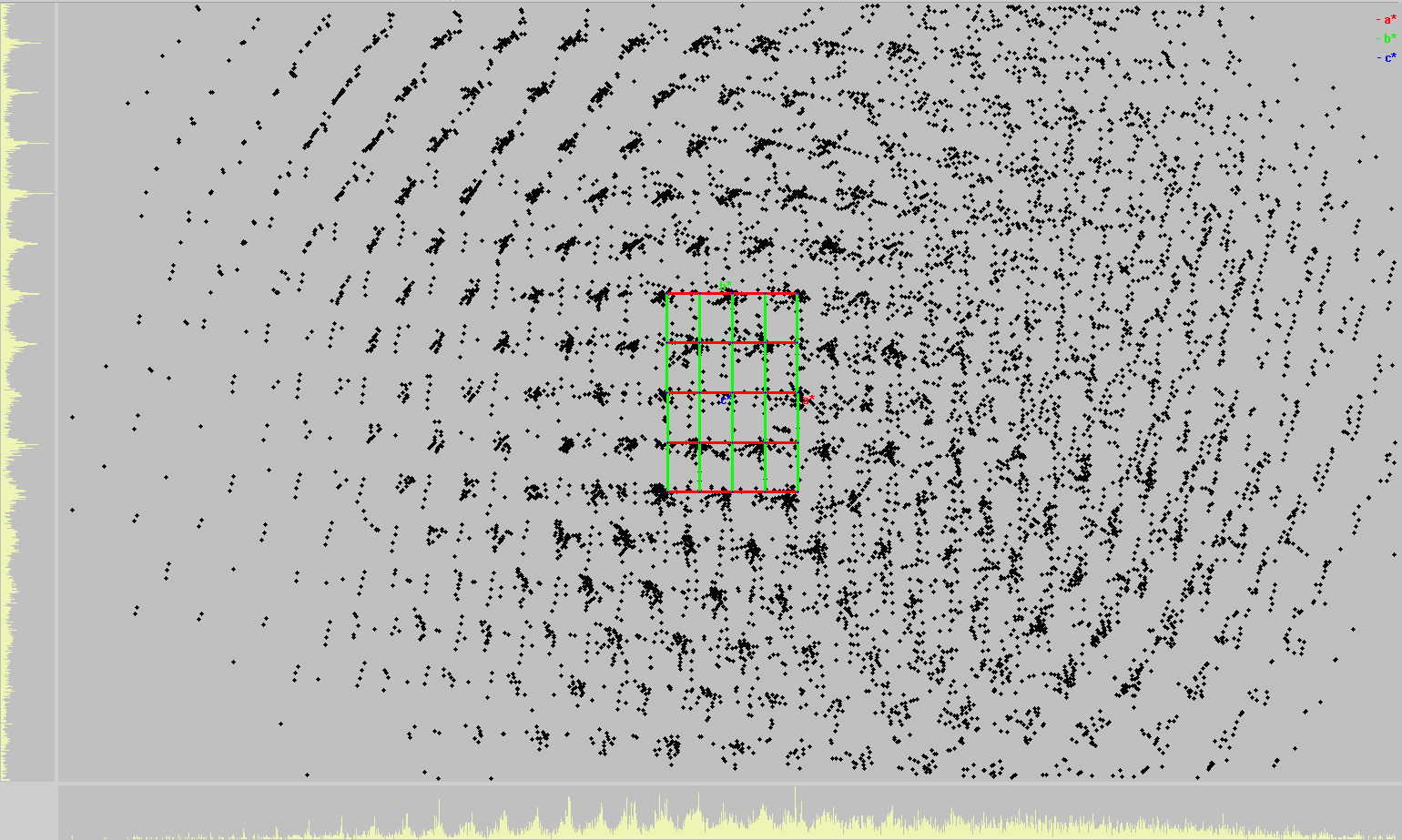

Supplement: Supplementary file 7 [file e-78-01097-sup7.png]
